# The Matched Education Dataset project: Methodology and lessons learned

**DOI:** 10.23889/ijpds.v8i2.2233

**Published:** 2023-09-14

**Authors:** Lucy Robinson

**Affiliations:** 1 Welsh Government, Cardiff, United Kingdom

## Objectives

There are separate data collections across education phases in Wales. Each use a different unique learner identifier. The matched education project used advanced data linking methods to produce a set of pseudo identifiers for each learner that can be matched back to the original datasets to undertake specific, anonymous analysis.

## Methods

The first phase of the project involved data cleaning, preparation, and the creation of new derived linking variables. The second phase of the project established the linking methodology, developing and making the most of advanced data linking techniques including frequency matching and phonetic string comparators. At each stage of the project the data linking was sequential, ranging from exact matching on all key variables to more fuzzy matching. During the code development the approach was uniquely tailored to each data set and constantly fine-tuned to ensure the highest possible match rate while reducing potential for false matches.

## Results

The resulting data sets are used in a number of ways for statistical and research purposes to support the formation of evidence-based policies. This includes research into raising the compulsory education age and the evaluation of learner journeys during the pandemic.

Robust linked data facilitates analysis examining the progression of learners through the education system in Wales. This has so far included published analysis on learner outcomes during the pandemic, and internal analysis looking at these outcomes by Free School Meal status.

Additionally, there is a broad scope of future analysis planned and the outputs of the matched education data set project will be used extensively in the evaluation of learner journeys post pandemic which will be inform Welsh policy.

## Conclusion

The matched education dataset project involved learning and upskilling in data linkage methodology and brought new data linking skills. We are keen to share lessons learned widely with the hope of improving the quality of data linkage projects and to reflect on the impact of data quality.

